# Efficacy and Safety of Continuous Subcutaneous Insulin Infusion vs. Multiple Daily Injections on Type 1 Diabetes Children: A Meta-Analysis of Randomized Control Trials

**DOI:** 10.4274/jcrpe.0053

**Published:** 2018-11-29

**Authors:** Yuan Qin, Lu-Hong Yang, Xiao-Li Huang, Xiao-Hong Chen, Hui Yao

**Affiliations:** 1 Huazhong University of Science and Technology, Tongji Medical College, Wuhan Children’s Hospital (Wuhan Maternal and Child Healthcare Center), Clinic of Children’s Genetic Metabolic Endocrine, Hubei Province, China

**Keywords:** Continuous subcutaneous insulin infusion, multiple daily injections, children, type 1 diabetes, meta-analysis

## Abstract

**Objective:**

This meta-analysis was performed to evaluate the efficacy and safety of continuous subcutaneous insulin infusion (CSII) vs. multiple daily injections (MDI) in children with type 1 diabetes.

**Methods:**

A literature search was conducted on databases including PubMed and Embase up to June 2017. The pooled weighted mean difference or risk ratio as well as 95% confidence intervals were calculated using RevMan 5.3 software.

**Results:**

Eight studies involving 310 children with type 1 diabetes were included. Results showed that HbA1c (%) was significantly lower (p=0.007) after CSII compared with MDI in children with type 1 diabetes. In addition, there was no significant difference between groups in HbA1c (%) change, total daily insulin doses, change of total daily insulin doses and incidence of ketoacidosis and severe hypoglycemia. However, subgroup analyses indicated that age, treatment duration and study design were influenced the efficacy of CSII and MDI in children with type 1 diabetes.

**Conclusion:**

CSII is associated with lower HbA1c levels in children with type 1 diabetes but appears to have no effect on insulin requirement or incidence of ketoacidosis and severe hypoglycemia.

**What is already known on this topic?**

A previous meta-analysis on children with type 1 diabetes indicated the advantages of continuous subcutaneous insulin infusion in blood glucose control. However, bias caused by age may exist.

**What this study adds?**

A better control of glycemia can be accomplished by continuous subcutaneous insulin infusion (CSII) compared with multiple daily injections (MDI) in children with type 1 diabetes aged ≤18 years old. The significantly reduced insulin requirement can be obtained after long term CSII treatment (12 months), compared with MDI. Age, treatment duration and study design are factors impacting the efficacy of CSII and MDI in children with type 1 diabetes.

## Introduction

Type 1 diabetes is caused by the immune system attacking and destroying the beta cells in the pancreas that produce insulin and commonly occurs in childhood with increasing incidence continuing in recent years (1). Multiple daily injection (MDI) treatment is the most widely used method of insulin administration for treating diabetes, which requires at least three or more injections a day. In recent years, to reduce the complications and to improve blood glucose control, continuous subcutaneous insulin infusion (CSII) has been used as a popular option for diabetes management, especially in preschool-aged children (2,3).

Recently, many meta-analyses were performed to compare MDI and CSII in adult patients with type 1 diabetes (4,5). In these studies, CSII was shown to have many advantages including improvement of blood glucose control, reduction of daily insulin requirement and increase of treatment satisfaction. In addition, a previous meta-analysis (6) of studies involving children with type 1 diabetes also indicated the advantages of CSII in blood glucose control. However, a study investigating patients older than 18 years (7) was included in that meta-analysis, so bias caused by age may have had an impact on the results. Thus, it is necessary to compare the efficacy and safety of CSII and MDI with studies comprising only children aged ≤18 years. In this present study, we also investigated the influence of treatment duration, age and study design on efficacy of CSII as compared to MDI.

## Materials and Methods

The methods used for this meta-analysis and generation of inclusion criteria were based on Preferred Reporting Items for Systematic Reviews and Meta-Analyses recommendations. Approval by a research ethics committee to conduct this meta-analysis was not required.

### Literature Search Strategy

Databases including PubMed and Embase were used for literature search up to June 2017, using the following keywords: [(insulin infusion) OR (insulin pump)] AND (children) AND [(diabetes) OR (diabetic)]. In addition, the references of relevant reviews were searched for additional studies.

### Inclusion and Exclusion Criteria

The following criteria were met for all included studies: (1) the study type was a randomized study; (2) subjects were children with type 1 diabetes aged ≤18 years old; (2) CSII was used for glucose control (experimental group) compared with conventional MDI (control group); (3) clinical outcomes included at least one of the following: HbA1c (%), insulin dose and some adverse events.

The studies were excluded if they were (1) duplicate publications, or (2) reviews, letters or comments.

### Data Extraction and Quality Assessment

The following data were recorded in a predesigned form: first author name, country, year, enrolled time, duration of diabetes, treatment duration, sample size, age, sex, treatment target, and outcomes. Data extraction was performed independently by two investigators. The quality of included studies was assessed by the Cochrane Collaboration’s tool for assessing risk of bias as described previously (8). For data extraction and quality assessment, differences were resolved by discussion to ensure consistency of evaluation.

### Statistical Analysis

The RevMan 5.3 software (RevMan 5.3, The Cochrane Collaboration, Oxford, UK) was used to perform this meta-analysis. The I-squared and Cochrane Q tests were used to assess the heterogeneity using p<0.1 or I^2^>50% indicating significant heterogeneity. An appropriate statistical model (fixed effect model or random effects model) was applied to pool the weighted mean difference (WMD) or risk ratio (RR) as well as the corresponding 95% confidence intervals (CIs), based on the results of heterogeneity test. The subgroup analysis was performed based on the age, treatment duration and study type. Publication bias was assessed using Egger’s and Begg’s Tests. For all these analyses, p<0.05 indicated statistical significance.

## Results

### Characteristics of Included Studies

**Figure 1:**
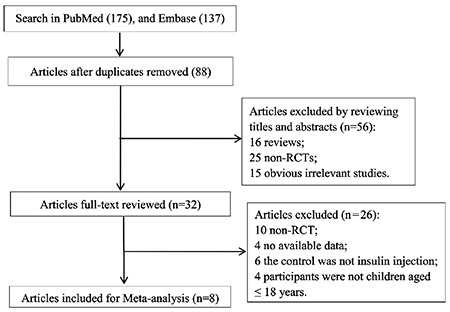
Flow diagram of the study selection process *RCT: randomised controlled trials*

After initial literature search, a total of 312 articles (PubMed: n=175, Embase: n=137) were identified. After excluding duplicates, 88 potentially relevant articles remained. Of these, 56 articles were excluded including 15 obvious irrelevant studies, 25 non-randomised controlled trials (non-RCTs) and 16 reviews. Then the remaining 32 articles were assessed by reading the full-text. Among them, 26 articles were excluded (10 were non-RCTs, four articles did not report available data, six articles did not use the insulin injection and four more studies enrolled some participants aged over 18 years). Finally, eight studies (9,10,11,12,13,14,15,16) were included in this analysis (Figure 1).

The characteristics of these studies are shown in Table 1. A total of 310 children with type 1 diabetes were included and reanalyzed in this meta-analysis. The duration of diabetes was longer than one year in all these patients. The publication year ranged from 2003 to 2014. There were six randomized control trials and two randomized crossover trials. The treatment durations ranged from 3.5 to 24 months. The bias risk assessment is shown in Table 2. No study applied or reported the blind method. Performance bias was avoided by crossover design only in the studies by Weintrob et al (12,13).

### Meta-analysis

**Figure 2:**
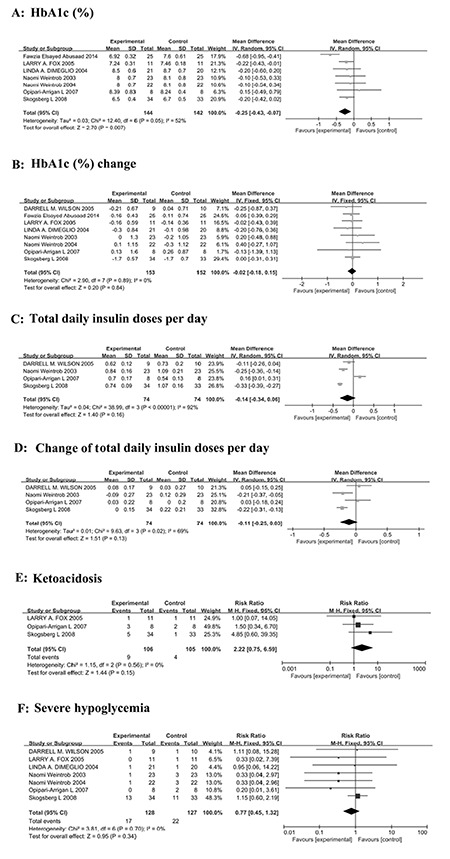
Forest plots for meta-analysis on HbA1c (%) (A), HbA1c (%) change (B), total daily insulin doses per day (C), change of total daily insulin doses per day (D) and incidence of ketoacidosis (E) and severe hypoglycemia (F)

All eight studies included in this analysis reported glucose control as the main outcome. As shown in Figure 2A, in children with type 1 diabetes, HbA1c (%) was significantly lower (WMD=-0.25, 95% CI=-0.43 to -0.07, p=0.007) after treatment by CSII as compared with MDI. However, the significant difference disappeared in the subgroup analyses (Table 3) by studies with crossover design (p=0.53) or in comparing prepubertal and pubertal patients of school age (p=0.05). Moreover, no significant difference was found in mean change of HbA1c (%) (mean difference from baseline to end of study) between the children treated with CSII and MDI in the overall analysis (WMD=-0.02, 95% CI=-0.18 to 0.15, p=0.84, Figure 2B) and in the subgroup analyses (p>0.05, Table 3).

As shown in Figure 2C, the total daily insulin doses were similar in diabetic children after treatment by CSII and MDI (WMD=-0.14, 95% CI=-0.34 to 0.06, p=0.16). The mean change of total daily insulin dose from baseline to the end of the study (mean difference from baseline to end of study) was also similar between CSII and MDI groups (WMD=-0.11, 95% CI=-0.25 to 0.03, p=0.13, Figure 2D). In the subgroup analyses, the results indicated that children with type 1 diabetes needed significantly less daily insulin doses after 12 months of CSII treatment as compared with MDI (WMD=-0.21, 95% CI=-0.36 to -0.05, p=0.009, Table 3).

As for adverse events, there was no significant difference in the incidence of ketoacidosis (RR=2.22, 95% CI=0.75-6.59, p=0.15, Figure 2E) and severe hypoglycemia (RR=0.77, 95% CI=0.45-1.32, p=0.34, Figure 2F) between the children treated with CSII and MDI. No inconsistent results for analysis of incidence of severe hypoglycemia were found in subgroup analysis (p>0.05, Table 3).

### Heterogeneity Results

In overall analyses, significant heterogeneity (p<0.1 or I^2^>50%) among studies was found in analyses for HbA1c (%), total daily insulin doses and change in total daily insulin doses. Therefore, the randomized effects model was applied to pool the data. Fixed effect model was used for other analyses (Figure 2). However, these significant heterogeneities were still absent (p>0.1 or I^2^=0%, Table 3) among studies in some subgroup analyses for HbA1c (%) (treatment duration, 3 or 3.5 months; study design, crossover design; age, prepubertal school aged and pubertal patients) and change of total daily insulin doses (treatment duration, 6 months). Thus, beside age, treatment duration and study design, there were other sources of heterogeneity.

### Publication Bias

No significant publication bias was found by Egger’s and Begg’s tests in this study (p>0.05).

## Discussion

In this study, significantly lower HbA1c (%) values were reported in the CSII group as compared with the MDI group. Moreover, subgroup analysis showed a significant difference between the groups after both three to three-and-a-half months and six months treatment. Indeed, many retrospectively or prospectively observational studies on the long term outcomes using HbA1c as the outcome measure in type 1 diabetic children (17,18), reported that CSII may have a significant better efficacy on glucose control after long term treatment. More studies should be performed to investigate the efficacy difference between long-term and short-term treatment. Our subgroup analysis also showed that study design may be a factor affecting the results, based on the subgroup analysis by study design for HbA1c (%). Lack of effect in RCTs suggests training in diabetes management may be main cause explaining CSII effects. In addition, the mean change of HbA1c (%) was similar among groups. The different baseline level or low number of studies may be the factors leading to the similar results between CSII and MDI groups. Furthermore, the effect of CSII on HbA1c (%) may be related to more diabetic education in children with diabetes and their families. The family or children treated by CSII may receive more diabetic education due to more opportunity to contact new treatment information and good economic incomes. More studies should be performed to investigate the impact of diabetic education level on CSII or MDI treatment efficacy.

However, based on the results of subgroup analyses, the advantage [as measured by reduction in HbA1c (%)] of CSII compared with MDI was just absent in prepubertal school aged and pubertal patients in this study (p=0.05). Thus, age may be a factor affecting the efficacy of CSII and MDI treatment for type 1 diabetes. The pathogenesis of type 1 diabetes is mainly related to immune system mediated cell injury in the pancreas. Significant heterogeneity (I^2^=70%, p=0.02) existed among the included studies on prepubertal school aged and pubertal patients. Compliance with therapy may be a factor influencing the results, which is notoriously poor among pubertal aged patients but may be improved using CSII whereas prepubertal and preschool children age more under the control of their patients. Thus, the results are conflicting. More studies should be performed to confirm the impact of age on efficacy of CSII and MDI.

In addition, the insulin requirement was reported to be significantly reduced after long-term (12 months) CSII treatment compared with MDI, but not after short-term treatment (six months), which is inconsistent with the previous meta-analysis (6). This previous meta-analysis included a study on type 1 diabetes patients aged 8-21 years old (7). The findings on adult patients (of ages over 18 years) in this series with type 1 diabetes may have led to a result in bias risk affecting the results on children. Thus, we only included studies with children aged ≤18 years old in this meta-analysis Moreover, we included more studies in this meta-analysis, such as Opipari-Arrigan et al (11), Abusaad (15) and Skogsberg et al (16). In addition, we performed the subgroup analyses by study design. The heterogeneity changes and inconsistent results between subgroup analyses and overall analyses indicated that age, study design and treatment duration may be sources of heterogeneity and factors impacting the efficacy of CSII and MDI in children with type 1 diabetes.

In addition, no significantly different incidence of complications, in particular ketoacidosis and/or severe hypoglycemia, were found in this meta-analysis. However, some previous observational studies indicated that the CSII could significantly reduce the incidence of severe hypoglycemic episodes compared with MDI after long term treatment (five years) (19). Thus, more studies with longer follow-up periods need to be performed to further compare the complications after CSII and MDI in children with type 1 diabetes and explore the factors influencing the safety of CSII and MDI in these children.

### Study Limitations

Firstly, the number of included studies and sample size were small. Secondly, significant heterogeneity was found among the results of the studies. Although the subgroup analyses were performed, the significant heterogeneity still existed in some subgroup analyses. In addition to differences in study design, age and treatment duration, some other confounding factors (such as sex, duration of diabetes, country and treatment target) may also be sources of this heterogeneity. With increase in duration of diabetes, there is more and more risk of ‘’burn-out’’ and noncompliance of patients, which will affect the efficacy of treatment for glycemic control. However, the data for duration of diabetes is inadequate in the studies analyzed to perform the subgroup analyses in this meta-analysis. Therefore, this factor (duration of diabetes) needs to be investigated in further studies. Thirdly, in addition to HbA1c (%), duration of blood glucose value at the target range is also a key index evaluating the efficacy of blood glucose control. However, there were not sufficient data to perform a subgroup analysis in this meta-analysis.

## Conclusions

In conclusion, CSII is associated with lower HbA1C levels in children with type 1 diabetes but may have no effect on insulin requirement and in reducing incidence of ketoacidosis and severe hypoglycemia. Age, treatment duration and study design may be the factors influencing the comparison results. Diabetic education level may be one of the important factors influencing treatment efficacy. More studies should be performed to investigate the impact of diabetic education level on CSII or MDI treatment efficacy.

## Figures and Tables

**Table 1:**
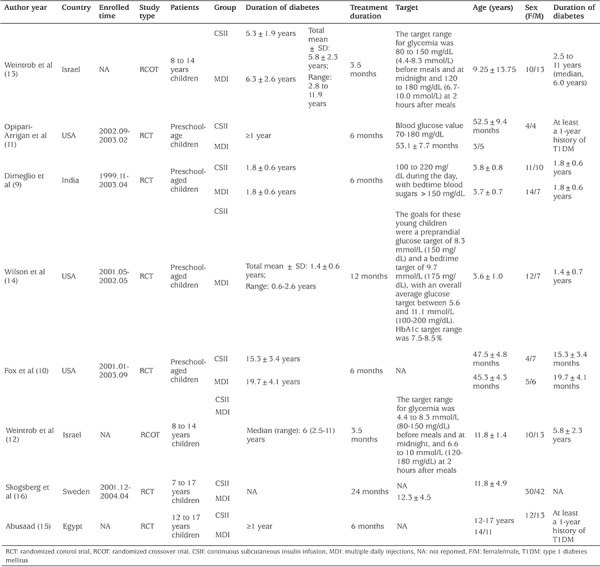
The characteristics of included studies

**Table 2:**
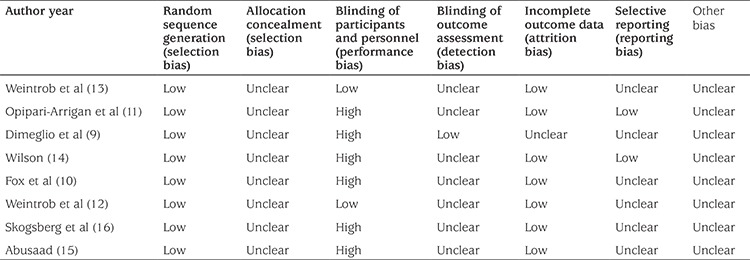
The assessment of bias risk of included studies

**Table 3:**
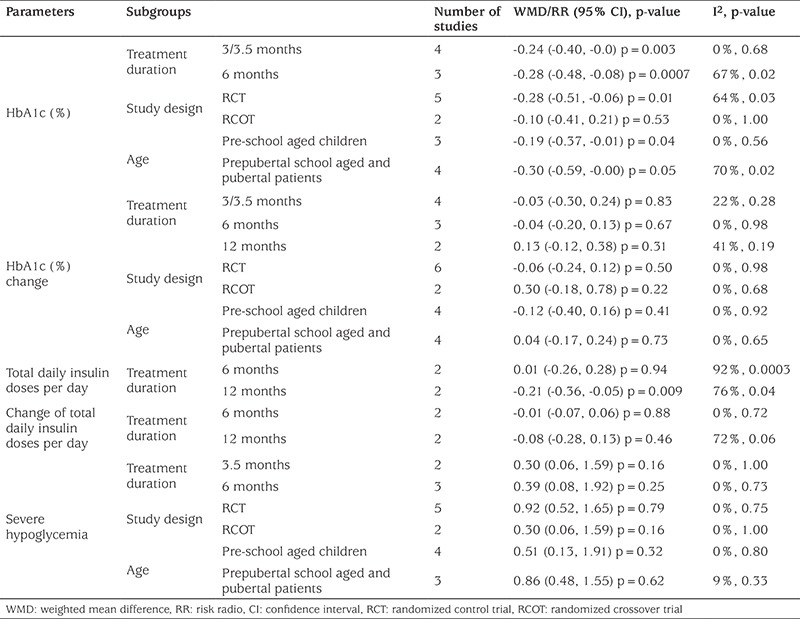
The results of subgroup analyses
